# Serological and RT-PCR evaluation of African yam bean (*Sphenostylis stenocarpa* (Hochst ex. A. Rich) Harms) accessions to viral resistance under field condition

**DOI:** 10.1038/s41598-024-59977-6

**Published:** 2024-04-27

**Authors:** Ihenacho Jeffrey, Iyabode Kehinde, Emily Ayo-John, Paul Bankole, Michael Abberton, P. Lava Kumar, Taofeek Adegboyega, Olaniyi Oyatomi

**Affiliations:** 1 Genetic Resources Center, International Institute of Tropical Agriculture, Ibadan, Nigeria; 2 Germplasm Health, Virology and Molecular and Diagnostics Unit, International Institute of Tropical Agriculture, Ibadan, Nigeria; 3 Department of Pure and Applied Botany, Federal University of Agriculture, Abeokuta, Ogun State Nigeria; 4 Department of Crop Protection, Federal University of Agriculture, Abeokuta, Ogun State Nigeria; 5 Biology Unit, Faculty of Science, Air Force Institute of Technology, PMB 2014, Nigerian Air Force Base, Kaduna, Kaduna State Nigeria; 6 Food Security and Safety Niche, Faculty of Natural and Agricultural Science, North-West University, Mmabatho, 2735 South Africa; 7 Department of Microbiology and Parasitology, School of Medicine and Pharmacy, University of Rwanda, Kigali, Rwanda

**Keywords:** African yam bean, ACP ELISA, RT-PCR, Viral infection, Disease incidence and severity, Microbiology, Plant sciences

## Abstract

African yam bean (AYB) (*Sphenostylis stenocarpa* (Hochst ex. A. Rich.) harms) an underutilized legume that produces nutritionally healthy seeds and tubers in some variety. The low yield of the crop is attributed to production constraints such as attacks by pest and disease-causing organisms such as fungi, bacteria and viruses. In this study, one hundred AYB accessions were evaluated for resistance to viral infection. The AYB accessions were planted using a randomized complete block design on the experimental field at the International Institute of Tropical Agriculture (IITA) Ibadan, Nigeria. Viral disease severity was assessed at 10, 12, 14, 16 and 18 weeks after planting (WAP) based on disease symptoms using disease severity index on visual scale of 1–5. Antigen–coated plate enzyme linked immunosorbent assay (ELISA) and reverse transcription polymerase chain reaction were used to index diseased leaf samples collected from the field. Result from five virus species (Cowpea mild mottle virus, Cowpea mottle virus, Southern bean mosaic virus, Cowpea mosaic virus and Bean common mosaic virus) were detected in few accessions while mixed infections were observed in some accessions. TSs-552, TSs-577, TSs-580, TSs-560 and TSs-600 were devoid of viruses and could be resistant. There were no significant differences at p < 0.05 in the mean disease incidence (DI) of viral diseases. However, at 18 weeks after planting, TSs-604 had the highest (100%) mean DI while TSs-584 had the lowest (13.33%) mean DI. Cluster analysis based on the AUDPC produced 6 main clusters, the clusters revealed grouping patterns in which AYB lines with similar resistance ratings were shown to form unique clusters. The information generated from this study will contribute to the development of strategies in the management of virus diseases infecting AYB.

## Introduction

African yam bean (*Sphenostylis stenocarpa* (Hochst ex. A. Rich.) harms) is a nutritionally important but neglected food crop with several benefits. The crop has the ability to produce bean seed in a pod with varying seed patterns and colours^1^. In addition to the seeds, farmers can also harvest tubers, which resemble sweet potato. The tubers mature in 5 to 8 months^2^. The edible root tubers are rich in nutrients suitable for human consumption^3^. One of the limiting factors to the production of African yam bean is it low grain yield when compared with other legumes^4^. Hence, the low yield of the crop is attributed to production constraints such as unavailability of improved seeds, poor farm practices, attack by pest and disease-causing organisms such as fungi, bacteria and which viruses pose a major concern to African yam bean production. Viral diseases are among the most important pathogens in agriculture and have the ability to cause great economic losses to farmers by affecting the yield quality of the crop^5,6^. Viruses pose serious risks and may restrict the international movement of improved or selected germplasm due to quarantine restrictions^7^. Viral infected plant produce little or no flower buds and pod^8^. Several viruses have been reported worldwide, of which two viruses have been identified serologically infecting AYB^9^. This includes cowpea mild mottle virus (CPMMV) and black eye cowpea mosaic virus (BCMV). Symptoms of viral diseases in AYB include mosaic, distortion, yellow chlorosis and stunting of the plant. Effective management of these viral diseases are quite important to improve yield of AYB. Conventional methods used by farmers to control viruses including broad-spectrum insecticides for the control of vectors that transmit the viruses are inadequate and not cost-effective to the farmers and not environmentally friendly. The use of host plant resistance is therefore considered the most economical and environmentally friendly approach in the management of viral diseases. This study was conducted to evaluate one hundred AYB accessions and to identify the associated viruses using serological and RT-PCR techniques. Detection of AYB virus will be important in developing measures to control the virus infecting AYB.

## Results

### Disease symptoms observed

**Figure 1:**
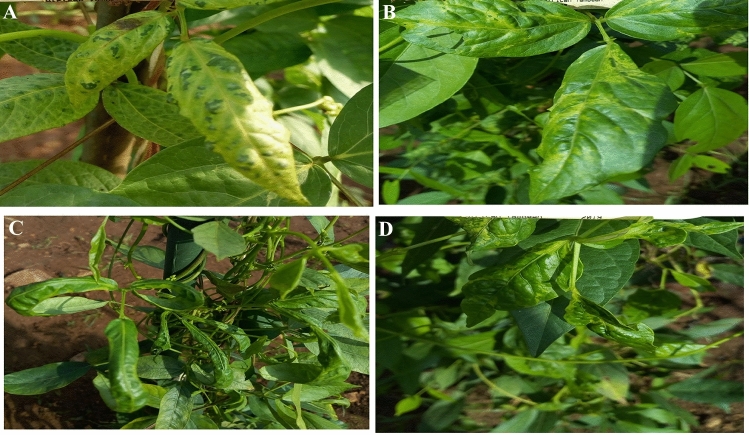
Viral disease symptoms observed in the open field; (**A**) Severe mosaic. (**B**) Mosaic and puckering and (**C**) Severe mosaic and leaf curl. (**D**) Mosaic and stunting.

**Figure 2:**
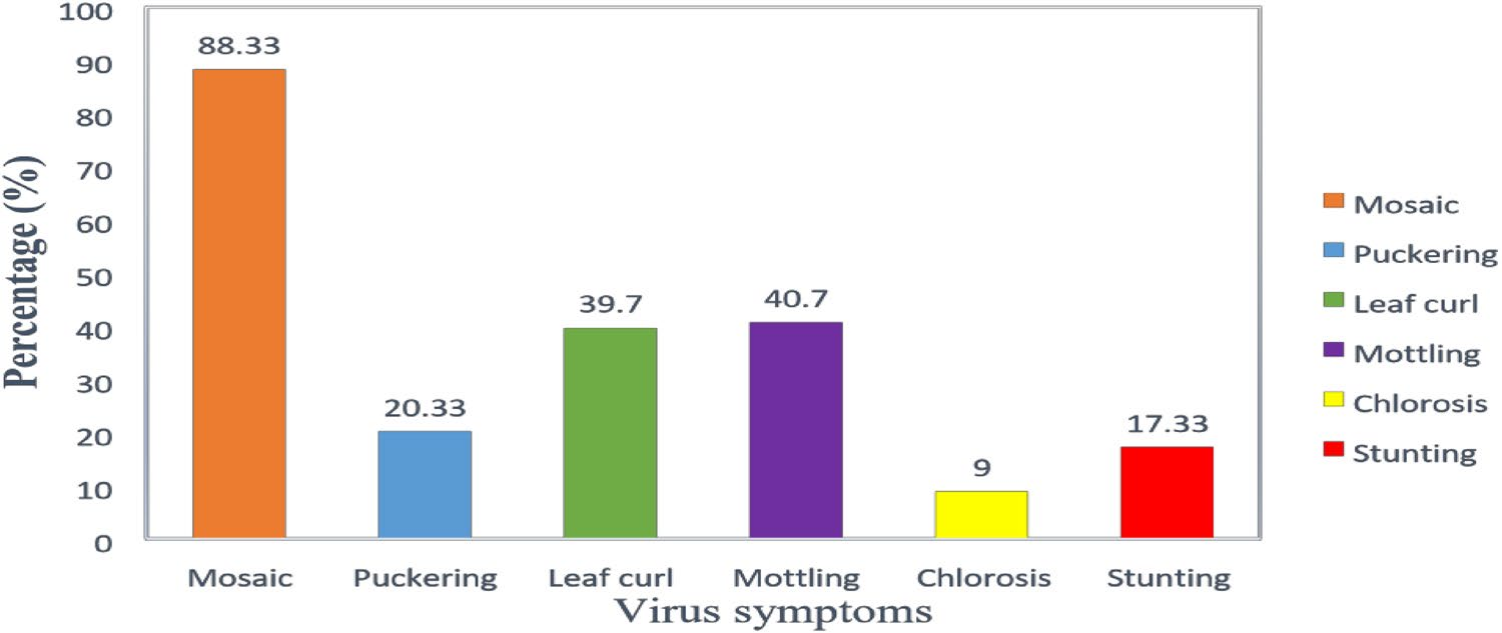
Viral symptoms observed during 2019/2020 planting season.

Virus and virus-like disease symptoms were observed on 100 AYB accessions on the field. The symptoms included different symptoms such as mosaic, puckering, stunting, leave curl, chlorosis and mottling (Fig. 1). Over 88.33% of the accessions showed mosaic followed by mottling (40.7%), Leaf curl (39.7%), puckering (20.3%), stunting (17.3%) whereas chlorosis was the least (9%) (Fig. 2).

### Incidence of viral diseases

Mean incidence of viral diseases on the 100 accessions of African yam bean is presented in Table 1. Generally, for all 100 African yam bean accessions, the incidence of viral diseases increased from 10 to 18 WAP, with overall mean incidences increasing from 27.01 to 60.39%. ANOVA showed no significant differences in the mean incidence of viral diseases during the 2019/2020 planting season. At the final observation (18 WAP), TSs-601 had the highest mean incidence (93.33%), while TSs-584 had the lowest mean incidence of 13.33%.

**Table 1:** Mean incidence of viral diseases among 100 AYB accessions showing the top 10 and bottom 5 ranked accessions during the 2019/2020 planting season.

Accession	10 WAP	12 WAP	14 WAP	16 WAP	18 WAP
Top 10 accessions					
TSs-604	63.33	65	66.67	78.33	100
TSs-601	33.33	41.67	41.67	63.33	93.33
TSs-533	60	66.67	66.67	66.67	86.67
TSs-535	66.67	66.67	66.67	73.33	86.67
TSs-547	53.33	53.33	53.33	66.67	86.67
TSs-553	73.33	73.33	80	80	86.67
TSs-555	33.33	53.33	60	73.33	86.67
TSs-556	53.33	66.67	80	80	86.67
TSs-567	33.33	33.33	66.67	66.67	86.67
TSs-541	36.67	45	61.67	68.33	83.33
Bottom 5 accession					
TSs-580	26.67	26.67	26.67	26.67	33.33
TSs-590	0	0	6.67	13.33	33.33
TSs-570	20	20	30	30	30
TSs-586	0	6.67	13.33	13.33	26.67
TSs-584	0	0	6.67	13.33	13.33
Grand mean	27.01	31.98	38.4	47.82	60.39
P-value	0.33	0.53	0.61	0.52	0.48

Key: *TSs* tropical *Sphenostylis stenocarpa*, *WAP* weeks after planting.

### The severity of viral diseases and area under disease progress curve

**Figure 3:**
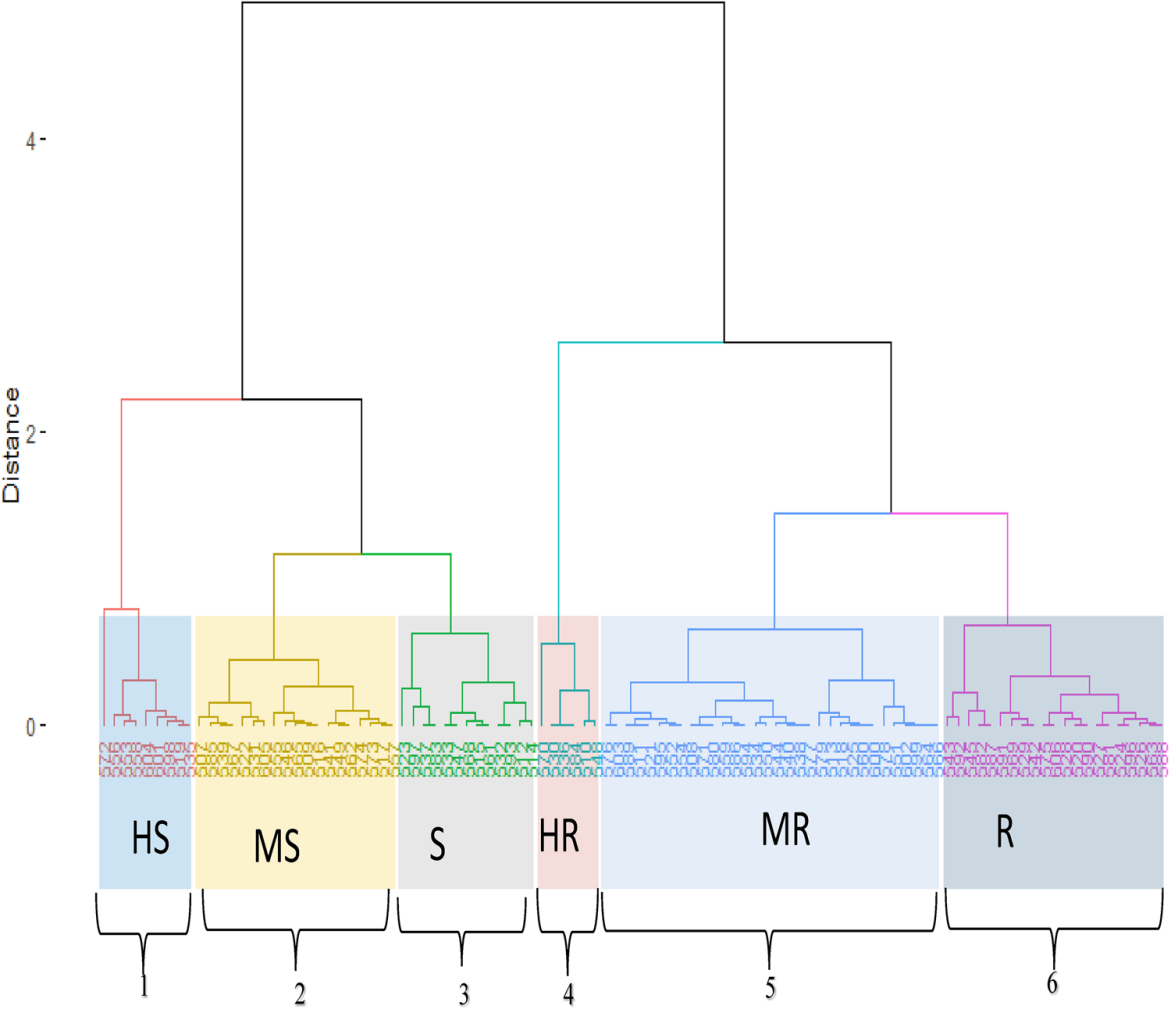
Cluster dendrogram of 100 accessions of AYB based on severity viral symptoms and AUDPC.

The results of cluster analysis performed based on the severity of the viral symptoms and area under disease progressive curves (AUDPC) are presented in Fig. 3 the 100 accessions were grouped into six major clusters. Cluster 1 has 8 accessions which are highly susceptible to the observed viral symptoms based on AUDPC. Cluster 5 comprises of 32 accessions that were all moderately resistant to viral symptoms.

### Viral detection by antigen-coated plate enzyme-linked immunosorbent assay (ACP-ELISA) and reverse transcriptase polymerase chain reaction (RT-PCR)

**Figure 4:**
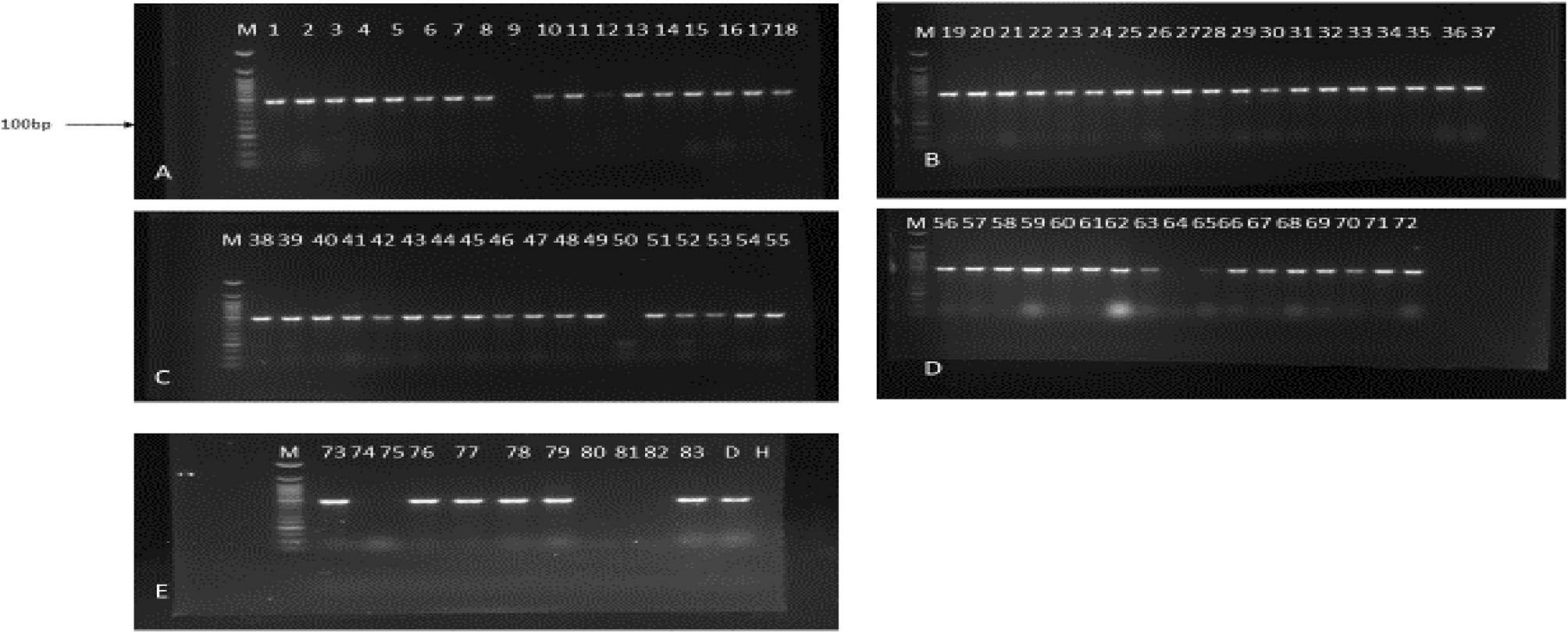
Agarose gel electrophoresis (2% agarose) of resolved amplified RT-PCR products of BCMV polyprotein gene (469 bp). Stained with ethidium bromide. Lane M: Marker 100 bp DNA; Lanes 1–18 test samples; Lanes 19–37 test samples; Lane 38–55 test samples; lane 56–72 test sample; lane 73–83 test samples, *H* healthy control, and *D* disease control.

Five virus species namely CPMMV, CPMoV, SBMV, CMV, and BCMV were detected in the African yam bean accessions using ACP-ELISA. RT-PCR detected BCMV in several accessions and CAbMV was not detected in the accession. Seventy-five sample out of the eighty symptomatic samples collected was infected with at least one of the five viruses (Table 2). It was observed that some viruses were associated with single or multiple infections in the plant samples. The AYB samples had a high prevalence of single virus infection compared with multiple virus infections. In single virus-infected leaf samples, BCMV was the most prevalent, infecting 73.75% of the samples tested, Co-infection by BCMV + CPMMV was observed in twelve African yam bean accessions. BCMV, CPMMV, and SBMV were observed in two AYB accession. BCMV + CPMMV + SBMV + CMV were observed in one accession of AYB; five accessions of AYB TSs-552, TSs-577, TSs-580, TSs560, and TSs600 were not positive for any of the four antibodies used in this study (Fig. 4).

**Table 2:** Viruses detected in different AYB accession by ACP ELISA and RT-PCR.

Virus detected	No of accession	accessions	ELISA and RT-PCR reaction
BCMV*	59	TSs-507, TSs-509, TSs-514, TSs-515, TSs-525, TSs-509, TSs-514, TSs-515, TSs-525, TSs-532, TSs-533, TSs-535, TSs-555, TSs-558, TSs-564, TSs-566, TSs-571, TSs-581, TSs-586, TSs-590, TSs-591, TSs-598, TSs-605, TSs-606, TSs-508, TSs-516, TSs-518, TSs-519, TSs-528, TSs-536, TSs-538, TSs-544, TSs-554, TSs-562, TSs-570, TSs-573, TSs-582, TSs-588, TSs-594, TSs-597, TSs-601, TSs-604, TSs-513, TSs-523, TSs-529, TSs-534, TSs-537, TSs-539, TSs-541, TSs-569, TSs-572, TSs-579, TSs-593, TSs-595, TSs-511, TSs-512, TSs-517, TSs-553, TSs-556, TSs-567, TSs-596, TSs-526, TSs-561	+ (BCMV was detected only by RT- PCR)
BCMV* + CPMMV	12	TSs-583, TSs-583, TSs-584, TSs-531, TSs-540, TSs-543, TSs-557, TSs-587, TSs-522, TSs-565, TSs-585, TSs-574	+
BCMV* + CPMMV, SBMV	2	TSs-575, TSs-592	+
BCMV* + CPMMV + SBMV + CMV + CMoV	1	TSs-549	+
CPMMV	1	TSs-530	+
CAbMV*		–	Not detected
–	5	TSs-552, TSs-577, TSs-580, TSs-560, TSs-600	No virus detected

*RT-PCR used for the detection of BCMV and CAbMV; all other viruses were detected using ACP-ELISA.

## Discussion

The success of disease resistance breeding relies on precise identification of pathogen and accurate screening of germplasm lines for a particular disease-causing pathogen. It is preferable to adopt disease management strategies including breeding resistant plants, limiting the movement of plant material, and searching for new variants to lessen the damage that plant-pathogenic viruses can cause^10,11^. This study was conducted to evaluate one hundred AYB accessions and to identify the associated viruses. The AYB accessions exhibited various symptoms, including leaf mosaic, mottling, chlorotic, leaf curl, puckering and chlorosis. The presence of these symptoms suggested that AYB was infected with viruses on the field. Several types of symptoms have been reported on virus infecting leguminous plant^12–14^. The symptoms observed in this study on AYB accessions were in consistent with the symptom types that has been previously reported on AYB^9^. The various African yam bean symptoms observed in the field may be due to factors such as the genotype and type of virus, the time of infection of the virus pathogen, mixed infections and environment. Four viruses infecting African yam bean was detected using ACP-ELISA namely CMV, SBMV, CMoV and CPMMV. These viruses can cause great damage on plant. RT-PCR was used for detecting the presence of BCMV. Bean common mosaic virus (BCMV) is one of the most prevalent and harmful viruses, affecting both cultivated and wild range of leguminous plant^15^. The viruses identified in this study are among the virus listed by Hughes et al. to be occurring in Nigeria^16^. Fifty-nine accessions out of the eighty symptomatic accessions were infected with BCMV. This suggest that BCMV may be the most common virus infecting African yam bean in the study area. One of the major causes of the reported low yield in AYB could be attributed to BCMV. The study also revealed mixtures of infection within the African yam bean accessions. Several reports have shown that multiple virus infections are usually associated with higher disease severity and yield reduction^17,18^. Virus-symptomatic plants not testing positive to any virus for which diagnostics were used in this study may be due to their low concentrations in the leaf samples^19^.

## Conclusions

This study demonstrated that leaf mosaic and mottling were the most common symptoms observed in AYB followed by chlorotic spots and the least was stunting. ACP-ELISA and RT-PCR tests detected five viruses in the accessions studied. In contrast, no virus was detected in accessions TSs-552, TSs-577, TSs580, TSs-560 and TSs-600 by ACP-ELISA and RT-PCR which may imply diseases resistance in these five African yam bean accessions for viruses tested. These accessions can be further screened against other African yam bean viruses to confirm their status. Confirmation of the resistance of these accessions may serve as a source of resistant genes for breeding work and planting material for farmers.

## Materials and methods

All methods used in this study were carried out in accordance with relevant guidelines and regulations.

**Figure 5:**
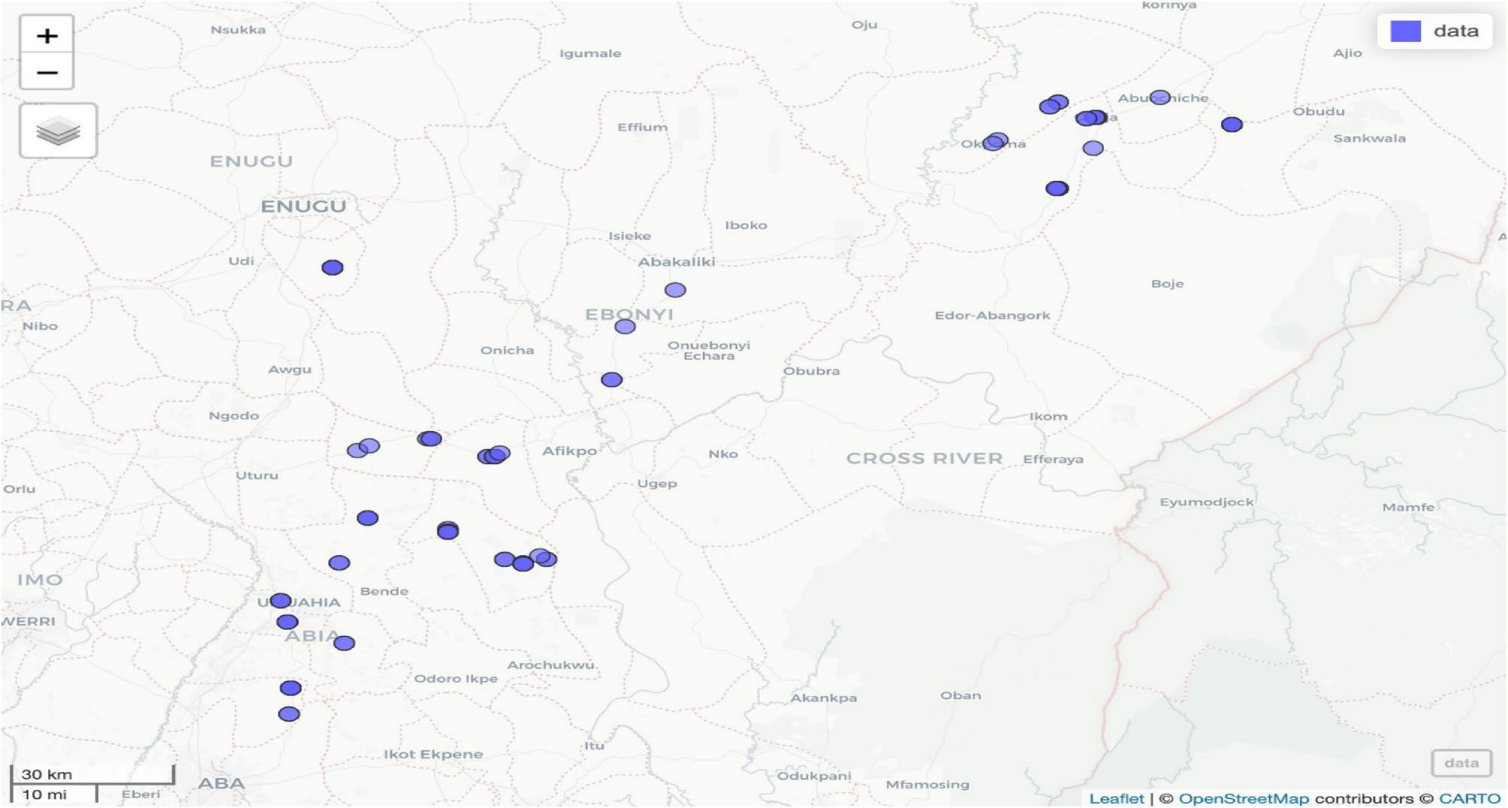
African yam bean accessions collection sites in Nigeria. Accessions from the same site are clustered together.

The research was conducted at the experimental field of the International Institute of Tropical Agriculture (IITA), Ibadan, Nigeria. One hundred accessions of African yam bean were obtained from the Genetic Resources Center of IITA Ibadan, Nigeria (Fig. 5). Seeds were planted on 5 m ridges, spaced 1 m apart. Each accession was planted on two rows at 1 m intra-row spacing. Initially, two seeds were planted per hill and later thinned to one plant per hill to give 10 plants per accession in each plot. The experiment was prepared in three replicates. Disease assessment was done 14 days interval starting from the fourth weeks after sowing (WAS) until senescence. (Table 3). Data on mean severity scores were used to calculate Area Under Disease Progress Curve for each of the African yam bean lines using the formula of Campbell and Madden^20^.

$$AUP{\text{C}}\sum\limits_{{t = {1}}}^{{n - {1}}} {0.{5}(x_{{i + {1}}} + x_{i} ) \, (t_{{i + {1}}} - ),}$$

where n was the total number of assessments, ti was the time of the ith assessment in days from the first assessment date, xi was percentage of disease severity at ith assessment.

**Table 3:** Severity scale and its description.

Scale	Symptoms description	Leaf area infected (%)
1	No virus symptoms	Disease free
2	Slightly mosaic on leaves, mild distortions, while the remaining parts of the leaves and leaflets appear green and healthy	1–25
3	Pronounced mosaic pattern on most leaves and distortion of the one-third of the leaflets	25–50
4	Severe mosaic, distortion of two-thirds of most leaves	50–75
5	Extensive mosaic and serious deformation of leaves, (or plant dead)	> 75

The severity of virus on the leaves was scored using the standard disease scale 1-5.

### Serological detection

Leaf samples collected from symptomatic and asymptomatic plants were tested with antigen-coated-plate ELISA (ACP-ELISA) using six polyclonal antibodies specific for legumes; Cowpea mild mottle virus (CPMMV), Cowpea mottle virus (CMV), Southern bean mosaic virus (SBMV), Cowpea mottle virus (CPMoV), Bean common mosaic virus (BCMV) and Cowpea Aphid-borne mosaic virus (CABMV). The ACP ELISA was carried out as described by Kumar^21^. African yam bean leaf samples were ground at a ratio of 0.1 g ml^–1^ (1:10 w/v) with a mortar and pestle in coating buffer (Na_2_CO_3_ 1.59 g, NaHCO_3_ 2.93 g and sodium diethyldithiocarbamate 10 g in 1 L of distilled water with pH 9.6). Hundred micro liters (100 ul) of p- nitrophenyl phosphate substrate was added to each well and incubated at room temperature for 1 h, and overnight at 4 °C to allow color development. Optical density values were read at 405 nm using a Bio-Rad microplate reader (ELx 800, Universal Microplate Reader). The result was considered positive when the value is greater or equal to twice the absorbance value of healthy control.

### Detection by reverse transcription polymerase chain reaction (RT-PCR)

Samples confirming virus positive were amplified by modified reverse transcriptase-polymerase chain reaction (RT-PCR) protocol using only two coat protein specific primers (Cowpea aphid-borne mosaic virus (CABMV) − 525bp and Bean common mosaic virus (BCMV)—469bp) RT-PCR was set up for BICMV and CABMV-positive samples in ACP-ELISA, using primers designed to coat protein as follows BCMVF3X 5′-ATGTGGTACAATGCTGTGAAG, BCMV B3X TTTCAGTATTCTCGCTGGTTG. CABMV coat protein gene, CABMV F3X 5′- GTACTCCAGTCTGATGGAAAGG, CABMV B3X GTCCGAGAAGTGGTGCATAA. Total nucleic acid was extracted from African yam bean leaf samples using modified Cetyltrimethyl Ammonium Bromide (CTAB) method^22^. The extracted RNA was used as template in RT-PCR to amplify 469 bp of the partial coat protein gene of BCMV and 525 bp corresponds to the CABMV coat protein. One step RT-PCR amplification of viral RNA strands were carried out. Amplification of BCMV RNA was done using 1 cycle of 44 °C for 30 min, 1 cycle of 95 °C for 1 min; 35 cycles of 94 °C for 1 min, 52 °C for 1 min, 72 °C for 1 min, 72 °C for 7 min as final extension and store forever at 4 °C. For CABMV amplification was carried out by 1 cycle of 42 °C for 30 min, 1 cycle of 94 °C for 5 min; 35 cycles of 94 °C for 30 s, 54 °C for 30 s, 72 °C for 30 s, 72 °C for 5 min as final extension and store forever at 4 °C. The amplification products were resolved on 1.5% agarose gel stained with GR green (5 µl/100 ml) and run in the electrophoretic tank containing TAE buffer pH 8.0 at 120 V for 40 min. The gel result was viewed under a UV transilluminator (EZ imager, Bio-Rad, Inc, USA).

### Data analysis

Data collected on viral disease incidence and severity were subjected to analysis of variance using SAS software with means separated by Duncan Multiple Range test at p ≤ 0.05.

## Data Availability

Data and materials are disclosed to manuscript. Further information on data and materials can be directed to the corresponding authors.

## Abbreviations

WAP: Weeks after planting
ACP-ELISA: Antigen–coated plate enzyme linked immunosorbent assay
AYB: African yam bean
IITA: International Institute of Tropical Agriculture (IITA)
AUDPC: Area under disease progressive curve
RT-PCR: Reverse transcription polymerase chain reaction
CTAB: Cetyltrimethyl ammonium bromide
CPMMV: Cowpea mild mottle virus
CMV: Cowpea mottle virus
SBMV: Southern bean mosaic virus
CPMoV: Cowpea mottle virus
BCMV: Bean common mosaic virus
CABMV: Cowpea Aphid-borne mosaic virus
TSs: Tropical Sphenostylis stenocarpa
DI: Disease incidence
