# Author Correction: Serological and RT-PCR evaluation of African yam bean (*Sphenostylis stenocarpa* (Hochst ex. A. Rich) Harms) accessions to viral resistance under field condition

**DOI:** 10.1038/s41598-024-66719-1

**Published:** 2024-07-10

**Authors:** Ihenacho Jeffrey, Iyabode Kehinde, Emily Ayo-John, Paul Bankole, Michael Abberton, P. Lava Kumar, Taofeek Adegboyega, Olaniyi Oyatomi

**Affiliations:** 1 Genetic Resources Center, International Institute of Tropical Agriculture, Ibadan, Nigeria; 2 Germplasm Health, Virology and Molecular and Diagnostics Unit, International Institute of Tropical Agriculture, Ibadan, Nigeria; 3 Department of Pure and Applied Botany, Federal University of Agriculture, Abeokuta, Ogun State Nigeria; 4 Department of Crop Protection, Federal University of Agriculture, Abeokuta, Ogun State Nigeria; 5 Biology Unit, Faculty of Science, Air Force Institute of Technology, PMB 2014, Nigerian Air Force Base, Kaduna, Kaduna State Nigeria; 6 Food Security and Safety Niche, Faculty of Natural and Agricultural Science, North-West University, Mmabatho, 2735 South Africa; 7 Department of Microbiology and Parasitology, School of Medicine and Pharmacy, University of Rwanda, Kigali, Rwanda

Correction to: *Scientific Reports* 10.1038/s41598-024-59977-6, published online 27 April 2024

The original version of this Article contained errors.

The name of author P. Lava Kumar was incorrectly given as Kumar Lava.

In addition, Ihenacho Jeffrey was incorrectly affiliated with ‘Germplasm Health, Virology and Molecular and Diagnostics Unit, International Institute of Tropical Agriculture, Ibadan, Nigeria’. The correct affiliations are listed below.

Genetic Resources Center, International Institute of Tropical Agriculture, Ibadan, Nigeria.

Department of Pure and Applied Botany, Federal University of Agriculture, Abeokuta, Ogun State, Nigeria.

The original Article has been corrected.

